# Supplementary material for: Comparison between Macro and Trace Element Concentrations in Human Semen and Blood Serum in Highly Polluted Areas in Italy
Source: Int J Environ Res Public Health. 2022 Sep 15;19(18):11635. doi: 10.3390/ijerph191811635 (PMC9517217; doi:10.3390/ijerph191811635)
Supplement: Supplementary file 1 [file ijerph-19-11635-s001.zip › ijerph-1876777-supplementary.pdf]

Supplementary Materials

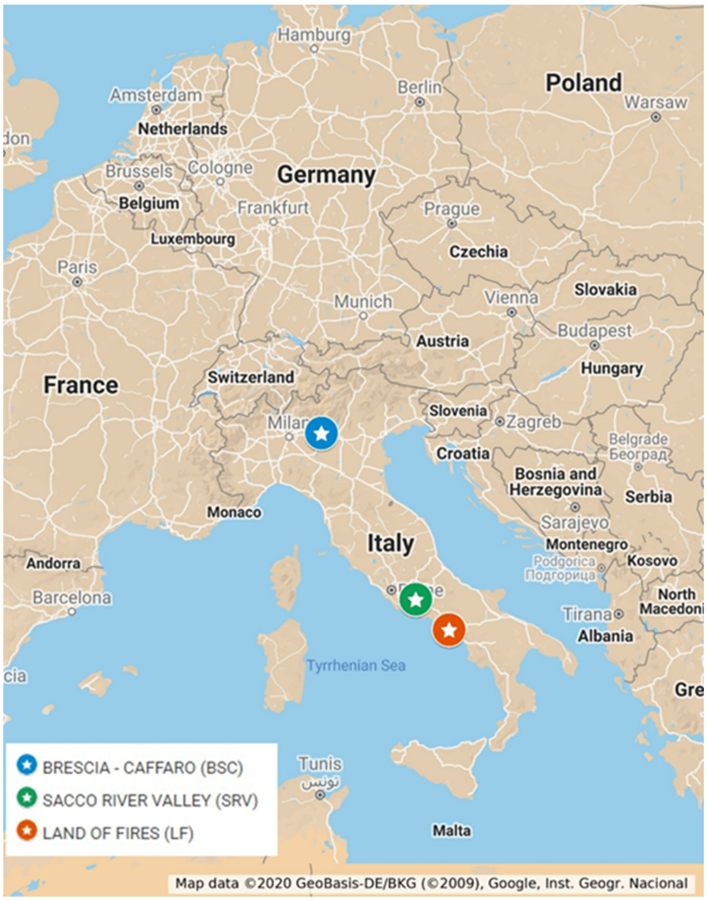

Figure S1. Map of the areas selected for the recruitment: SIN Brescia-Caffaro area (northern Italy, blue circle), SIN Sacco River Valley (central Italy, green circle) and the northern area of city of Naples, “Land of Fires” (southern Italy, orange circle).

Table S1. General characteristics of the study population reported for each area as mean value ± standard deviation.

	**BSC**	**LF**	**SRV**	**ρ value[1]**
**N**	144	137	51	-
**Age (year)**	20.0 ± 1.2	18.8 ± 1.3	18.2 ± 0.5	0.0001
**Weight (Kg)**	70.6 ± 9.0	71.6 ± 9.7	73.4 ± 8.7	n.s.
**Height (cm)**	177.3 ± 5.9	175.6 ± 6.5	177.3 ± 6.8	n.s.
**BMI (Kg m^{-2})**	22.4 ± 2.3	23.2 ± 2.7	23.4 ± 3.0	0.0318
**Abdominal circumference (cm)**	83.5 ± 5.8	88.2 ± 7.2	83.0 ± 7.7	0.0001

[1] p-values were calculated with the Kruskal-Wallis Test; n.s.: not significant p value (>0.05)

Table S2. Lifestyle factors (PREDIMED and IPAQ scores) and semen quality parameters of subjects in each area reported as mean value ± standard deviation

	BSC	LF	SRV	ρ value[1]
**PREDIMED score**	6.9 ± 2.1	7.4 ± 2.3	7.2 ± 2.5	n.s.
**IPAQ score (Met)**	2380.4 ± 2105.7	2858.9 ± 3219.5	3414.6 ± 2729.0	n.s.
**Volume (mL)**	2.9 ± 1.4	2.5 ± 1.1	2.9 ± 1.6	n.s.
**pH**	8.1 ± 0.4	7.9 ± 0.1	7.8 ± 0.2	0.0001
**Spermatic concentration (10^6 ml^{-1})**	67.3 ± 45.9	47.6 ± 32.7	52.3 ± 37.9	0.001
**Total Motility (%)**	39.8 ± 20.0	42.7 ± 26.2	26.7 ± 21.6	0.0002
**Progressive motility (%)**	27.1 ± 18.1	28.9 ± 20.5	16.8 ± 15.6	0.0008
**Cells with normal morphology (%)**	6.4 ± 4.4	7.1 ± 7.3	5.0 ± 3.2	n.s.

[1] p-values were calculated with the Kruskal-Wallis Test; n.s.: not significant p value (>0.05)

Table S3. Limit of detection (LOD) and limit of quantification (LOQ) in the final sample expressed in μg/L.

	Unit	Ca	Mg	Na	K	Cu	Fe	Mn	Ni	Se	Zn	Al	As	Ba
**LOD**	μg/L	200	200	200	200	4.4	3.4	0.4	4.2	1.1	1.8	1.2	0.2	6.6
**LOQ**	μg/L	500	500	500	500	11.0	8.5	1.0	10.5	2.8	4.5	3.0	0.5	16.5
	**Unit**	**Be**	**Cd**	**Co**	**Cr**	**Hg**	**Li**	**Pb**	**Rb**	**Sb**	**Sn**	**Sr**	**U**	**V**
**LOD**	μg/L	2.6	0.2	0.4	0.8	0.2	0.4	0.1	3.0	0.2	0.2	0.2	0.2	1.8
**LOQ**	μg/L	6.5	0.5	1.0	2.0	0.5	1.0	0.2	7.5	0.5	0.5	0.5	0.5	4.5

Table S4. Blood serum sample below limit of detection (LOD) expressed in %.

	Whole cohort		BSC		LF		SRV	
	**n**	**%**	**n**	**median**	**n**	**median**	**n**	**median**
**Ca**	332	0	144	0	137	0	51	0
**Mg**	332	0	144	0	137	0	51	0
**Na**	332	0	144	0	137	0	51	0
**K**	332	0	144	0	137	0	51	0
**Cu**	332	0	144	0	137	0	51	0
**Mn**	332	13,9	144	13,9	137	0	51	51,0
**Ni**	332	100	144	100	137	100	51	100
**Se**	332	0	144	0	137	0	51	0
**Zn**	332	0	144	0	137	0	51	0
**Al**	332	100	144	100	137	100	51	100
**As**	332	0	144	0	137	0	51	0
**Ba**	332	21,4	144	10,4	137	24,8	51	43,1
**Be**	332	78,0	144	76,4	137	72,3	51	98,0
**Cd**	332	56,6	144	72,9	137	37,2	51	62,8
**Co**	332	100	144	100	137	100	51	100
**Cr**	332	100	144	100	137	100	51	100
**Hg**	332	43,4	144	34,0	137	35,8	51	90,2
**Li**	332	2,5	144	5,6	137	0	51	0
**Pb**	332	4,2	144	5,6	137	4,4	51	0
**Rb**	332	0	144	0	137	0	51	0
**Sb**	332	12,0	144	4,9	137	17,5	51	17,6
**Sn**	332	15,4	144	33,3	137	0,73	51	3,9
**Sr**	332	0	144	0	137	0	51	0
**U**	332	41,9	144	50,0	137	29,9	51	51,0
**V**	332	100	144	100	137	100	51	100

Table S5. Semen sample below limit of detection (LOD) expressed in %.

	Whole cohort		BSC		LF		SRV	
	**n**	**%**	**n**	**median**	**n**	**median**	**n**	**median**
**Ca**	268	0	113	0	100	0	55	0
**Mg**	268	0	113	0	100	0	55	0
**Na**	268	0	113	0	100	0	55	0
**K**	268	0	113	0	100	0	55	0
**Cu**	268	0	113	0	100	0	55	0
**Mn**	268	0	113	0	100	0	55	0
**Fe**	268	0	113	0	100	0	55	0
**Ni**	268	40,7	113	3,5	100	93,0	55	21,8
**Se**	268	0	113	0	100	0	55	0
**Zn**	268	0	113	0	100	0	55	0
**Al**	268	100	113	100	100	100	55	100
**As**	268	0,37	113	0,88	100	0	55	0
**Ba**	268	23,9	113	56,6	100	0	55	0
**Be**	268	72,0	113	65,5	100	67,0	55	94,6
**Cd**	268	94,8	113	87,6	100	100	55	100
**Co**	268	100	113	100	100	100	55	100
**Cr**	268	100	113	100	100	100	55	100
**Hg**	268	33,3	113	20,4	100	19,0	55	70,9
**Li**	268	0,37	113	0,88	100	0	55	0
**Pb**	268	4,1	113	9,7	100	0	55	0
**Rb**	268	0	113	0	100	0	55	0
**Sb**	268	60,4	113	41,6	100	66,0	55	89,1
**Sn**	268	3,0	113	0,88	100	6,0	55	1,8
**Sr**	268	0	113	0	100	0	55	0
**U**	268	97,0	113	96,5	100	96,0	55	100
**V**	268	100	113	100	100	100	55	100

Table S6. Precision, accuracy and recovery data evaluated on blood serum samples.

	Unit	C_{obs}	C_{spike}	CV%	Rec %
**Ca**	mg/L	187	-	5.6	-
**Mg**	mg/L	35.2	-	9.0	-
**Na**	mg/L	227	-	2.8	-
**K**	mg/L	4392	-	4.8	-
**Cu**	µg/L	60.4	100	20	81
**Mn**	µg/L	-	100	28	92
**Ni**	µg/L	8.8	100	22	112
**Se**	µg/L	3.6	100	14	80
**Zn**	µg/L	37.4	100	7.0	96
**Al**	µg/L	-	100	11	104
**As**	µg/L	-	100	25	85
**Ba**	µg/L	-	100	13	116
**Be**	µg/L	-	100	19	98
**Cd**	µg/L	-	100	21	93
**Co**	µg/L	-	100	11	82
**Cr**	µg/L	-	100	12	107
**Hg**	µg/L	0.8	100	27	95
**Li**	µg/L	12.2	100	16	116
**Pb**	µg/L	-	100	17	93
**Rb**	µg/L	11.1	100	23	116
**Sr**	µg/L	1.3	100	25	118
**V**	µg/L	-	100	14	119

[1] C_{obs}, observed concentration in unfortified blood serum sample

[2] C_{spike}, known added spiked concentration

[3] CV%, mean value of coefficient of variation

[4] Rec%, mean recovery based on spiked concentration

Table S7. Precision, accuracy and recovery data evaluated on semen samples.

	Unit	C_{obs}	C_{spike}	CV%	Rec %
**Cu**	μg/L	8.0	100	24	90
**Mn**	μg/L	0.9	100	25	86
**Ni**	μg/L	-	100	2	105
**Se**	μg/L	3.7	100	10	88
**Zn**	mg/L	4.6	-	5	-
**Al**	μg/L	-	100	8	115
**As**	μg/L	3.2	100	15	85
**Ba**	μg/L	-	100	5	118
**Be**	μg/L	-	100	5	94
**Cd**	μg/L	-	100	4	82
**Co**	μg/L	-	100	3	114
**Cr**	μg/L	-	100	10	108
**Fe**	μg/L	165	100	6	120
**Hg**	μg/L	0.5	-	22	-
**Li**	μg/L	1.4	100	24	118
**Pb**	μg/L	-	100	4	85
**Rb**	μg/L	75	100	5	116
**Sr**	μg/L	3.5	100	9	114
**V**	μg/L	-	100	9	114

[1] C_{obs}, observed concentration in unfortified blood serum sample

[2] C_{spike}, known added spiked concentration

[3] CV%, mean value of coefficient of variation

[4] Rec%, mean recovery based on spiked concentration

Table S8. Recovery data evaluated on CRMs.

				This work	
		**Unit**	**Certified value** [1]	**Mean value** [2]	**Recovery %**[3]
**BCR - 304**	**Ca**	mmol/L	2.201 ± 0.019	2.496 ± 0.255	113
**BCR - 304**	**Mg**	mmol/L	1.85 ± 0.03	1.67 ± 0.15	90
**BCR - 304**	**Li**	mmol/L	0.985 ± 0.029	1.126 ± 0.105	114
**BCR - 638**	**Se**	μg/L	104 ± 7	111 ± 18	107
**BCR - 638**	**Zn**	μg/L	1430 ± 210	1602 ± 273	112

[1] unweighed mean value and expanded uncertainty; [2] unweighed mean value and standard deviation of 5 replicates; [3] unweighed mean value of 5 replicates

Table S9. Reference Values and biomonitoring data expressed as μg/L for macro and essential trace elements in blood serum.

	**WHO** [1,2]		**ISS 10/22** [3]		**ISS 11/9** [4]		**SIVR** [5]	
	**range**	**year**	**range**	**year**	**range[a,b]**	**year**	**range[b]**	**year**
**Ca**	-	-	52577 - 70632	2005	-	-	-	-
**Mg**	-	-	14643 - 20255	2005	-	-	-	-
**Cu**	800 – 1200[c]	1996	601 - 1373	1990	-	-	500 - 1250	2005
	-	-	648 - 1301	2005	-	-	600 – 1600[d]	2011
**Mn**	0.5 - 1	1996	0.3 - 0.9	1990	0.47 - 1.38	-	0.1 - 1.1	2005
	-	-	0.31 - 1.02	2005	-	-	-	-
**Ni**	< 1 - 2	1996	0.10 - 1.25	2005	0.09 - 0.95	-	0.1 - 1	2005
	-	-	0.26 - 0.75	2006	-	-	-	-
**Se**	75 - 120	1996	56 -105	1990	-	-	50 - 130	2005
	39 - 197	2005	-	-	-	-		
**Zn**	800 - 1100	1996	587 - 1215	1990	-	-	600 - 1080	2005
	-	-	597 - 1028	2005	-	-	800 - 1600	2011

[a] data referred to adults aged between 18 and 35; [b] data expressed as 5° - 95° percentiles; [c] referred to males; [d] referred to adults

Table S10. Reference Values and biomonitoring data expressed as μg/L for additional and not essential trace elements in blood serum.

	**WHO** [1,2]		**ISS 10/22** [3]		**ISS 11/9** [4]		**SIVR** [5]	
	**range**	**year**	**range**	**year**	**range[a,b]**	**year**	**range[b]**	**year**
**Al**	-	-	0.03 – 7.5	1990	-	-	1.0 – 6.0	2005
	-	-	0.43 – 5.29	2005	-	-	-	-
**As**	< 1 - 5	1996	-	-	< 0.15 - 2.80	-	-	-
**Ba**	-	-	0.32 - 1.37	2005	-	-	0.2 - 1.2	2005
**Be**	-	-	0.03 - 0.27	1990	< 0.022 - 0.086	-	0.06 - 0.25	2005
	-	-	0.06 - 0.43	2005	-	-	-	-
**Cd**	0.1 - 0.2	1996	0.04 - 0.36	1990	0.032 - 0.272	-	0.1 - 0.15	2005
	-	-	0.03 - 0.20	1990	-	-	-	-
**Co**	-	-	0.08 – 0.4	1990	0.074 – 0.485	-	0.05 – 0.30	2005
	-	-	0.06 – 0.42	2005	-	-	-	-
**Cr**	0.14 – 0.15	1996	0.04 – 0.41	1990	0.054 – 0.292	-	0.1 – 0.20	2005
	-	-	0.07 – 0.28	2005	-	-	-	-
**Hg**	< 1	1996	0.6 - 3.8	1990	0.14 - 1.88	-	0.2 - 1.5	2005
**Li**	0.2 - 0.8	1996	0.36 - 2.20	2005	-	-	-	-
**Pb**	< 1	1996	0.1 - 0.5	1990	0.05 - 0.68	-	0.05 - 0.35	2005
	-	-	0.20 - 0.98	2005	-	-	0.01 - 0.25[c]	2011
**Rb**	0.1 - 0.2	1996	78 - 317	1990	-	-	-	-
**Sb**	-	-	0.02 - 0.22	2005	0.028 - 0.340	-	-	-
**Sr**	-	-	23.0 - 61.5	2005	-	-	-	-
**V**	0.1 - 1.0	1996	0.07 - 1.1	1990	0.020 - 0.115	-	0.03 - 0.1	-
	-	-	0.03 - 0.11	2005	-	-	-	-

[a] data referred to adults aged between 18 and 35; [b] data expressed as 5° - 95° percentiles; [c] referred to plasma

Table S11. Schematic representation of trace elements found in human semen and their functions.

Elements	Role / Findings	Ref
**Ca**	Steroidogenesis; Acrosome reaction; Hyperactivation; Sperm quality; Chemotaxis	[6]
**Cu**	Sperm quality	[6–9]
**Fe**	Co-factor of Catalase	[7,9,10]
**K**	Sperm quality; Sperm capacitation	[6]
**Mg**	Spermatogenesis; Sperm quality	[6,9]
**Mn**	Sperm quality	[6]
**Na**	Spermatogenesis; Acrosome reaction; Sperm quality; Sperm capacitation	[6]
**Se**	Spermatogenesis; Sperm quality	[6,7]
**Zn**	Steroidogenesis; Spermatogenesis; Testicular development; Sperm quality; Sperm capacitation; Chemotaxis	[6–9,11]
**As**	Spermatogenesis ↓ ; Sperm maturation ↓ ; Sperm motility ↓	[12]
**Ba**	Sperm motility ↓	[13]
**Be**	Chromosomial aberrations ↑ ; Sperm abnormalities ↑	[14]
**Cd**	Testicular morphology ↓ ; Spermatogenesis ↓; Sperm Quality ↓; Sperm Morphology ↓	[8]
**Li**	Sperm motility ↓	[15]
**Hg**	Testicular morphology ↓ ; Spermatogenesis ↓; Sperm Quality ↓; Sperm motility ↓ ; Sperm Morphology ↓	[16,17]
**Ni**	Sperm motility ↑	[18]
**Pb**	Testicular morphology ↓ ; Spermatogenesis ↓; Sperm Quality ↓; Sperm Morphology ↓	[8,19]
**Sr**	Sperm motility ↑ ; Sperm capacitation ↑ ; Acrosome reaction ↑	[20]
**V**	Sperm motility ↓ ; Sperm capacitation ↓	[19]

An up arrow (↑) represents an increment of that function, while a down arrow (↓) represents a decrease of that function.

Table S12. Ratio between Zn and Cu in blood serum.

	**WRC**	**BSC**	**LF**	**SRV**
**Zn/Cu Serum**	1,43	1,64	1,32	1,15

References

1. *Trace Elements in Human Nutrition and Health*; World Health Organization, Food and Agriculture Organization of the United Nations, International Atomic Energy Agency, Eds.; World Health Organization: Geneva, 1996; ISBN 978-92-4-156173-0.
2. *Global Health Risks: Mortality and Burden of Disease Attributable to Selected Major Risks*; World Health Organization, Ed.; World Health Organization: Geneva, Switzerland, 2009; ISBN 978-92-4-156387-1.
3. Alimonti, A.; Bocca, B.; Mattei, D.; Pino, A. Biomonitoraggio Della Popolazione Italiana per l'esposizione Ai Metalli: Valori Di Riferimento 1990-2009. Roma:Istituto Superiore Di Sanità (Rapporti ISTISAN 10/22).

4. Alimonti, A.; Bocca, B.; Mattei, D.; Pino, A. Programma per Il Biomonitoraggio Dell'esposizione Della Popolazione Italiana (PROBE): Dose Interna Dei Metalli. Roma:Istituto Superiore Di Sanità (Rapporti ISTISAN 11/9 IT). Available online: http://old.iss.it/binary/publ/cont/11_9IT_web.pdf (accessed on 15 September 2020).

5. SIVR - Società Italiana Dei Valori Di Riferimento Available online: http://www.sivr.it/documenti.htm (accessed on 20 December 2020).

6. Mirnamniha, M.; Faroughi, F.; Tahmasbpour, E.; Ebrahimi, P.; Harchegani, A.B. An Overview on Role of Some Trace Elements in Human Reproductive Health, Sperm Function and Fertilization Process. *Reviews on Environmental Health* **2019**, *34*, 339–348, doi:10.1515/reveh-2019-0008.

7. Nenkova, G.; Petrov, L.; Alexandrova, A. Role of Trace Elements for Oxidative Status and Quality of Human Sperm. *Balkan Med J* **2017**, *34*, 343–348, doi:10.4274/balkanmedj.2016.0147.

8. Sun, J.; Yu, G.; Zhang, Y.; Liu, X.; Du, C.; Wang, L.; Li, Z.; Wang, C. Heavy Metal Level in Human Semen with Different Fertility: A Meta-Analysis. *Biol Trace Elem Res* **2017**, *176*, 27–36, doi:10.1007/s12011-016-0804-2.

9. Hashemi, M.M.; Behnampour, N.; Nejabat, M.; Tabandeh, A.; Ghazi-Moghaddam, B.; Joshaghani, H.R. Impact of Seminal Plasma Trace Elements on Human Sperm Motility Parameters. *Romanian Journal of Internal Medicine* **2018**, *56*, 15–20, doi:10.1515/rjim-2017-0034.

10. Rubio-Riquelme, N.; Huerta-Retamal, N.; Gómez-Torres, M.J.; Martínez-Espinosa, R.M. Catalase as a Molecular Target for Male Infertility Diagnosis and Monitoring: An Overview. *Antioxidants* **2020**, *9*, 78, doi:10.3390/antiox9010078.

11. Verze, P.; Cai, T.; Lorenzetti, S. The Role of the Prostate in Male Fertility, Health and Disease. *Nat Rev Urol* **2016**, *13*, 379–386, doi:10.1038/nrurol.2016.89.

12. Kim, Y.-J.; Kim, J.-M. Arsenic Toxicity in Male Reproduction and Development Available online: http://www.ksdb.org/archive/view_article?pid=dr-19-4-167 (accessed on 18 December 2020).

13. Sukhn, C.; Awwad, J.; Ghantous, A.; Zaatari, G. Associations of Semen Quality with Non-Essential Heavy Metals in Blood and Seminal Fluid: Data from the Environment and Male Infertility (EMI) Study in Lebanon. *J Assist Reprod Genet* **2018**, *35*, 1691–1701, doi:10.1007/s10815-018-1236-z.

14. Fahmy, M.A.; Hassan, N.H.A.; Farghaly, A.A.; Hassan, E.E.S. Studies on the Genotoxic Effect of Beryllium Chloride and the Possible Protective Role of Selenium/Vitamins A, C and E. *Mutation Research/Genetic Toxicology and Environmental Mutagenesis* **2008**, *652*, 103–111, doi:10.1016/j.mrgentox.2007.12.009.

15. In Vitro Effects of Lithium on Human Sperm Motility. In Proceedings of the International Institute of Chemical, Biological & Environmental Engineering June 5-6, 2015 Istanbul (Turkey); International Institute of Chemical, Biological & Environmental Engineering, June 5 2015.

16. Bjørklund, G.; Chirumbolo, S.; Dadar, M.; Pivina, L.; Lindh, U.; Butnariu, M.; Aaseth, J. Mercury Exposure and Its Effects on Fertility and Pregnancy Outcome. *Basic & Clinical Pharmacology & Toxicology* **2019**, *125*, 317–327, doi:https://doi.org/10.1111/bcpt.13264.

17. Martinez, C.S.; Escobar, A.G.; Torres, J.G.D.; Brum, D.S.; Santos, F.W.; Alonso, M.J.; Salaices, M.; Vassallo, D.V.; Peçanha, F.M.; Leivas, F.G.; et al. Chronic Exposure to Low Doses of Mercury Impairs Sperm Quality and Induces Oxidative Stress in Rats. *J Toxicol Environ Health A* **2014**, *77*, 143–154, doi:10.1080/15287394.2014.867202.

18. Bian, J.; Shi, X.; Li, Q.; Zhao, M.; Wang, L.; Lee, J.; Tao, M.; Wu, X. A Novel Functional Role of Nickel in Sperm Motility and Eukaryotic Cell Growth. *J Trace Elem Med Biol* **2019**, *54*, 142–149, doi:10.1016/j.jtemb.2019.04.017.

19. Bae, J.-W.; Im, H.; Hwang, J.-M.; Kim, S.-H.; Ma, L.; Kwon, H.J.; Kim, E.; Kim, M.O.; Kwon, W.-S. Vanadium Adversely Affects Sperm Motility and Capacitation Status via Protein Kinase A Activity and Tyrosine Phosphorylation. *Reproductive Toxicology* **2020**, *96*, 195–201, doi:10.1016/j.reprotox.2020.07.002.

20. Okada, K.; Palmieri, C.; Della Salda, L.; Vackova, I. Viability, Acrosome Morphology and Fertilizing Capacity of Boar Spermatozoa Treated with Strontium Chloride. *Zygote* **2008**, *16*, 49–56, doi:10.1017/S0967199407004479.
